# Complications related to intraoperative transesophageal echocardiography in liver transplantation

**DOI:** 10.1186/s40064-015-1281-3

**Published:** 2015-09-04

**Authors:** Sher-Lu Pai, Stephen Aniskevich, Neil G. Feinglass, Beth L. Ladlie, Claudia C. Crawford, Prith Peiris, Klaus D. Torp, Timothy S. Shine

**Affiliations:** Department of Anesthesiology, Mayo Clinic, 4500 San Pablo Road South, Jacksonville, FL 32224 USA

**Keywords:** Coagulopathy, End-stage liver disease, Esophageal varices, Variceal bleed

## Abstract

**Purpose:**

Intraoperative transesophageal echocardiography (TEE) has commonly been used for evaluating cardiac function and monitoring hemodynamic parameters during complex surgical cases. Anesthesiologists may be dissuaded from using TEE in orthotopic liver transplantation (OLT) out of concern about rupture of esophageal varices. Complications associated with TEE in OLT were evaluated.

**Methods:**

We retrospectively reviewed charts and TEE videos of all OLT cases from January 2003 through December 2013 at Mayo Clinic (Jacksonville, Florida).

**Results:**

Of the 1811 OLTs performed, we identified 232 patients who underwent intraoperative TEE. Esophageal variceal status was documented during presurgical esophagogastroduodenoscopy in 230 of the 232 patients. Of these, 69 (30.0 %), had no varices; 113 (49.1 %), 41 (17.8 %), and 7 (3.0 %) had grades I, II, and III varices, respectively. Two patients (0.9 %) had no EGD performed because of acute liver failure. During OLT, 1 variceal rupture (0.4 %) occurred after placement of an oral gastric tube and TEE probe; the patient required intraoperative variceal banding. Most patients had preexisting coagulopathy at the time of probe placement. The mean (SD) laboratory test results were as follows: prothrombin time, 21.7 (6.6) seconds; international normalized ratio, 1.9 (1.3); partial thromboplastin time, 43.8 (13.3) seconds; platelet, 93.7 (60.8) × 1000/μL; and fibrinogen, 237.8 (127.6) mg/dL.

**Conclusion:**

TEE was a relatively safe procedure with a low incidence of major hemorrhagic complications in patients with documented esophagogastric varices and coagulopathy undergoing OLT. It appeared to effectively disclose cardiac information and allowed rapid reaction for proper patient management.

## Background

Intraoperative transesophageal echocardiography (TEE) has become an important part of an anesthesiologist’s armamentarium for evaluating cardiac function and monitoring hemodynamic parameters during complex surgical cases. Patients undergoing orthotopic liver transplantation (OLT) may present with cardiac dysfunction, either from preexisting coronary artery disease or associated liver pathophysiology (Carey et al. 1995; Zetterman et al. 1998). End-stage liver disease is often associated with a hyperdynamic state, resulting in reduced systemic venous resistance and increased cardiac output. Cirrhotic cardiomyopathy can cause hemodynamic changes, diastolic and systolic dysfunction, electrophysiologic abnormalities, and segmental wall motion abnormalities (Fouad and Yehia 2014; Matsumori 2005; Teragaki et al. 2003; Omura et al. 2005; Therapondos et al. 2004), which might be assessed using TEE.

Intraoperative cardiovascular instability may be exacerbated by major blood loss. In the pre-anhepatic phase, hypotension may result from large fluid shifts after ascitic fluid removal or from decreased venous return caused by surgical manipulation of the liver. Cross-clamping the inferior vena cava or even using an inferior vena cava–sparing piggyback technique (Lerut et al. 1989) can decrease venous return in the anhepatic phase, regardless of the availability of venovenous bypass (Burtenshaw and Isaac 2006). Cardiac instability during reperfusion is common because of the immediate decreased venous return and cardiac output. TEE can help assess the myocardial response to inotropes and the changes in preload and afterload after reperfusion. TEE can also identify cardiac pathologies and hemodynamic conditions that would be otherwise unknown without the visualization of the myocardial function (Markin et al. 2015).

Intraoperative TEE has been proven safe and efficacious during cardiac surgery (Reeves et al. 2013), with rates of major bleeding complications ranging from 0.03 to 0.8 % (Hahn et al. 2013). Its use has become more common in OLT because it provides real-time visualization of cardiac function. The guidelines published by the American Society of Echocardiography and the Society of Cardiovascular Anesthesiologists in 2013 stated that intraoperative TEE is generically indicated for “noncardiac surgery when patients have known or suspected cardiovascular pathology which may impact outcomes.” (Hahn et al. 2013) However, TEE probe insertion in the presence of the coagulopathy common to end-stage liver disease can result in esophageal injury and variceal hemorrhage. Esophageal varices, coagulopathy, thrombocytopenia, and recent upper gastrointestinal bleed are all listed as relative contraindications to TEE in the same published guidelines (Hahn et al. 2013). A limited number of retrospective reviews have evaluated the safety of intraoperative TEE in OLT and have suggested that TEE is a relatively safe method of monitoring intraoperative cardiac function, with rates of major hemorrhagic complications that range from none to 0.9 % in the overall OLT population and 2.8 % in patients with Model for End-Stage Liver Disease scores of 25 or higher (Burger-Klepp et al. 2012; Suriani et al. 1996; Myo Bui et al. 2015; Markin et al. 2015). The goal of this study was to investigate the complications associated with the use of intraoperative TEE during OLT at our institution, adding new data to the open discussion regarding the safety of TEE in the OLT patient population.

## Results

**Fig. 1:**
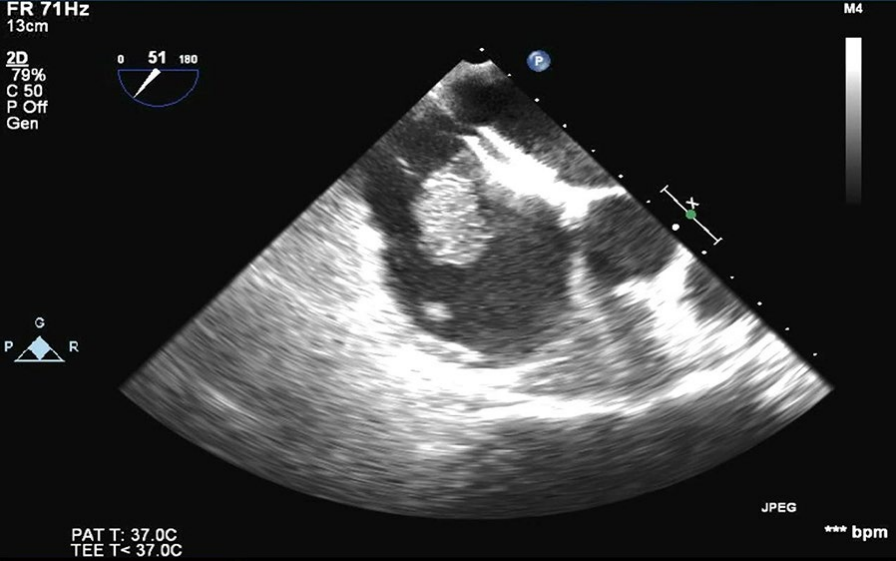
Intracardiac thrombus

**Fig. 2:**
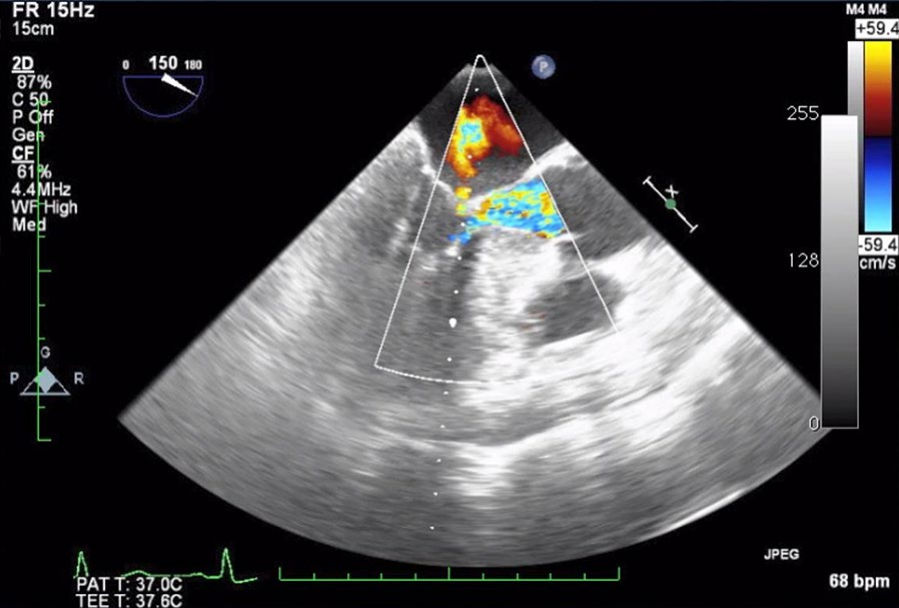
Left ventricular outflow obstruction with secondary left ventricular turbulence and mitral regurgitation

Of the 1811 patients undergoing OLT during the study period, 232 patients (12.8 %) had intraoperative TEE examinations, with a mean and median of 21 TEE examinations per year. The primary causes of end-stage liver disease of the study cohort are listed in Table 1. The most common indication for intraoperative TEE was hemodynamic monitoring, which was deemed beneficial by the anesthesiologists at their discretion. For hemodynamic monitoring, the TEE probe was inserted at the beginning of the procedure, after induction of general anesthesia. The second most common indication was emergent intraoperative TEE placement after a patient presented with hemodynamic instability or cardiac arrest. For these patients, TEE provided visual confirmation of intracardiac thrombosis (n = 4; Fig. 1) and dynamic left ventricular outflow tract obstruction (LVOTO) with systolic anterior motion of the mitral valve (n = 2; Fig. 2). The indications for intraoperative TEE are listed in Table 2. In total, 7 anesthesiologists performed the TEE examinations during the study period; of these, 2 had advanced perioperative echocardiography training and 5 had basic perioperative echocardiography training.

**Table 1:** Causes of end-stage liver disease requiring transplantation (N = 232)

Cause	No. of patients (%)
Acetaminophen toxicity	4 (1.7)
α-1 antitrypsin	2 (0.9)
Amyloidosis	2 (0.9)
Autoimmune hepatitis	8 (3.4)
Budd–Chiari syndrome	3 (1.3)
Cryptogenic cirrhosis	28 (12.1)
Alcoholic cirrhosis	29 (12.5)
Alcoholic cirrhosis + HCC	2 (0.9)
Alcoholic cirrhosis + nonalcoholic steatohepatitis	2 (0.9)
HCC	6 (2.6)
HCC + nonalcoholic steatohepatitis	2 (0.9)
Hepatitis A	1 (0.4)
Hepatitis B	3 (1.3)
Hepatitis B + HCC	2 (0.9)
Hepatitis C	53 (22.8)
Hepatitis C + alcoholic cirrhosis	10 (4.3)
Hepatitis C + HCC	13 (5.6)
Hepatitis C + alcoholic cirrhosis + HCC	1 (0.4)
Metastatic carcinoma to the liver	2 (0.9)
Nonalcoholic steatohepatitis	24 (10.3)
Polycystic liver or kidney disease	5 (2.2)
Primary biliary cirrhosis	10 (4.3)
Primary sclerosing cholangitis	10 (4.3)
Other	10 (4.3)

*HCC* hepatocellular carcinoma

**Table 2:** Indications for intraoperative transesophageal echocardiography (N = 232)

Indication	No. of patients (%)
Combined CABG and OLT	2 (0.9)
Hemodynamic instability	32 (13.8)
Hemodynamic monitoring	122 (52.6)
Known history	
Abnormal stress test	11 (4.7)
Abnormal ventricular wall motion	5 (2.2)
Aortic root dilatation, severe	1 (0.4)
Aortic stenosis, moderate and severe	7 (3.0)
Cardiac arrhythmia	12 (5.2)
Cardiac amyloidosis	1 (0.4)
Congestive heart failure or coronary artery disease	13 (5.6)
Flail mitral valve	1 (0.4)
Intracardiac thrombus	1 (0.4)
Left ventricular outflow tract obstruction	1 (0.4)
Mitral regurgitation, severe	1 (0.4)
Myocardial infarct, remote	7 (3.0)
Pericardial effusion	3 (1.3)
Portopulmonary hypertension	2 (0.9)
Systolic anterior motion of the mitral valve	5 (2.2)
Ventricular or atrial septal defect	5 (2.2)

*CABG* coronary artery bypass graft and *OLT* orthotopic liver transplantation

Of the 232 TEE examinations, 55 (23.7 %) had archived documentation of transgastric views, whereas the remainder were limited to the midesophageal view. The coagulation laboratory profile of these patients immediately preceding TEE probe insertion was noted (Table 3). Most patients had preexisting coagulopathy at the time of probe placement. One case had documentation describing a difficult insertion, which prompted the anesthesiologist to withdraw the probe and cancel the intraoperative TEE examination.

**Table 3:** Coagulation laboratory parameters of liver transplant patients immediately before intraoperative transesophageal echocardiography probe insertion

Characteristic	Mean (SD)	Median (IQR)
Prothrombin time (s)	21.7 (6.6)	20.0 (17.6–24.7)
International normalized ratio	1.9 (1.3)	1.7 (1.4–2.2)
Partial thromboplastin time (s)	43.8 (13.3)	41.6 (36.7–47.0)
Platelet (×1000/μL)	93.7 (60.8)	76.5 (56.0–107.0)
Fibrinogen (mg/dL)	237.8 (127.6)	207.0 (150.0–313.0)

*IQR* interquartile range

Esophageal variceal status had been documented by EGD in 230 of the 232 patients with intraoperative TEE (Table 4). The patients with a history of upper gastrointestinal tract bleeding and banding for treatment of esophageal varices are described in Table 5. Nearly all grade II and all grade III esophageal varices were banded during the transplant perioperative evaluation. Two patients (0.9 %) did not have EGD performed before OLT because of acute liver failure.

**Table 4:** Esophageal variceal grading by preoperative esophagogastroduodenoscopy in liver transplant recipients (N = 230)

Grade	No. of patients (%)
None	69 (30.0)
I	113 (49.1)
II	41 (17.8)
III	7 (3.0)

**Table 5:** Patients with history of UGI bleeding and banding for treatment of esophageal varices (N = 232)

	Total patients, no. (%)	Patients with intraoperative UGI bleeding after TEE placement, no. (%)
History of UGI bleeding		
Yes	44 (19.0)	1 (0.43)^a^
No	188 (81.0)	0 (0)
History of banding		
Yes	39 (16.8)	1 (0.43)
No	193 (83.2)	0 (0)

*TEE* transesophageal echocardiography and *UGI* upper gastrointestinal tract

^a^The same patient who had intraoperative UGI bleeding after TEE probe placement also had UGI bleeding with a subsequent variceal banding treatment

One patient had variceal rupture during OLT that occurred after placement of an orogastric tube and TEE probe (bleeding was observed in the oropharynx immediately after the insertion of the probe). Grade II bleeding esophageal varices were successfully banded during 2 preoperative EGD examinations performed 12 and 6 months before surgery. The patient again underwent EGD at 5 months and 2 weeks preoperatively; neither examination showed gastric varices and only trace esophageal varices were observed. The intraoperative upper endoscopy showed actively bleeding esophageal varices close to the gastroesophageal junction, where the gastroenterologist had successfully placed 4 bands to control the bleeding. Intraoperative coagulation parameters were as follows: PT, 16.7 s; PTT, 24.7 s; INR, 1.3; platelet count, 141 × 1000/μL; and fibrinogen, 342 mg/dL. No other complications (e.g., dental injury, oropharyngeal trauma, esophageal or gastric mucosa injury) were indicated.

In the remaining 1579 OLT patients without intraoperative TEE, 1 had an intraoperative variceal bleed without insertion of an orogastric tube or TEE probe in the esophagus. Coagulation parameters at the time of bleeding were as follows: PT, 33.6 s; PTT, 53.7 s; INR, 3.2; platelet count, 69 × 1000/μL; and fibrinogen, 94 mg/dL. The patient had grade I esophageal varices and gastric fundal varices. A Minnesota tube was placed by the gastroenterologist in the operating room with endoscopic guidance. The gastric balloon was insufflated and pulled against the gastroesophageal junction to stop the bleeding.

No dental injury, oropharyngeal trauma, esophageal or gastric mucosal injury, or death caused by TEE was found through our retrospective review of the immediate 24 h after the insertion of TEE probe.

## Discussion

In our series, intraoperative TEE appeared to be relatively safe, with a low incidence of major hemorrhagic complications in patients with documented esophageal varices and coagulopathy. It was associated with an esophageal hemorrhage rate of 0.4 % and no deaths. No dental injury, oropharyngeal trauma, and esophageal or gastric mucosa injury was observed in the perioperative period. TEE permitted the intraoperative diagnosis of intracardiac thrombosis in 4 patients and LVOTO in 2 patients. Although uncommon, both conditions can be potentially lethal if not diagnosed and treated in an appropriate and timely fashion. Only 23.7 % of TEE examinations were performed with transgastric views, but midesophageal views were still useful for monitoring hemodynamic and cardiac function.

In our patients with intracardiac thrombosis, clear visualization of the thrombi by intraoperative TEE prompted either intentionally sustained coagulopathy or rapid administration of heparin or tissue plasminogen activator to promote thrombolysis and decrease the risk of the clot propagation into the pulmonary vasculature (Lerner et al. 2005). Although intracardiac thrombosis is a rare event during OLT, its early diagnosis may be crucial for survival (Peiris et al. 2015).

In the 2 patients with LVOTO found after liver reperfusion, hemodynamic instability did not improve with vasopressor administration and volume resuscitation. The pulmonary artery catheter readings were relatively unchanged with central venous pressure, pulmonary capillary wedge pressure, systolic pulmonary artery pressure, and systemic venous resistance in the period preceding hypotension. Only diastolic pulmonary artery pressure became elevated during hemodynamic instability. A TEE probe was inserted and showed dynamic LVOTO with systolic anterior motion of mitral valve. Because TEE images allowed direct visualization of the ventricular walls, the anesthesiologists were able to immediately discontinue epinephrine, start β-blocking medications, and use phenylephrine as the only vasoactive agent.

TEE initially was used reluctantly during OLT because of the potential for variceal rupture (De Wolf 1999). Per the practice guidelines for perioperative TEE published by the American Society of Anesthesiologists and the Society of Cardiovascular Anesthesiologists Task Force in 2010 (Practice guidelines for perioperative transesophageal echocardiography. An updated report by the American Society of Anesthesiologists and the Society of Cardiovascular Anesthesiologists Task Force on transesophageal echocardiography 2010), esophageal varices are not an absolute contraindication to TEE. The guidelines recommend using TEE in patients with esophageal disease if the expected benefit outweighs the potential risk and appropriate precautions are taken (Practice guidelines for perioperative transesophageal echocardiography. An updated report by the American Society of Anesthesiologists and the Society of Cardiovascular Anesthesiologists Task Force on Transesophageal Echocardiography 2010). Studies have reported that TEE significantly improved the intraoperative management of patients undergoing OLT (Suriani et al. 1996; Rafferty et al. 1993). One study, although not conducted during intraoperative OLT procedures, showed that “TEE without transgastric views can be performed without serious complications in patients with grade I or II esophageal varices who have not experienced recent variceal hemorrhages”(Spier et al. 2009).

Two patients in our review had documented variceal rupture. One presented immediately after placement of the TEE probe, whereas the other patient did not receive any instrumentation or manipulation of the esophagus. Spontaneous variceal rupture can be caused solely by increased portal pressure after clamping the portal system at the start of the anhepatic phase (Boin et al. 2004). Variceal hemorrhage and esophageal complications can occur after insertion of an orogastric tube for stomach decompression (Taniai et al. 2000; Casavilla et al. 1991; Stevenson et al. 1992). We hypothesize that clamping of the portal system at the start of the anhepatic phase caused portal pressure to increase, leading to spontaneous rupture of the gastric varices in the patient without intraoperative TEE.

Although the incidence rate of variceal bleeding or esophageal injury from intraoperative TEE probe insertion was low in our cohort, the risks and benefits should still be diligently assessed for each patient. When performing intraoperative TEE during OLT, we suggest having at least one assistant who monitors the patient’s vital signs, administers medications, suctions secretions, and otherwise attends to the patient’s needs; this assistant may be extremely valuable, considering the significant workload and attention required for this patient population. When TEE examinations are performed by experienced operators with strict vigilance, they have an acceptably low risk of complications (Practice guidelines for perioperative transesophageal echocardiography. An updated report by the American Society of Anesthesiologists and the Society of Cardiovascular Anesthesiologists Task Force on Transesophageal Echocardiography 2010; Kallmeyer et al. 2001; Daniel et al. 1991). In summary, methods to decrease the likelihood of variceal rupture are listed in Table 6.

**Table 6:** Methods to decrease the risk of variceal rupture during intraoperative TEE in liver transplantation

1. Allow TEE examinations to be performed by experienced operators with strict vigilance (Practice guidelines for perioperative transesophageal echocardiography. An updated report by the American Society of Anesthesiologists and the Society of Cardiovascular Anesthesiologists Task Force on Transesophageal Echocardiography 2010; Kallmeyer et al. 2001; Daniel et al. 1991)
2. Obtain a gastroenterology consultation for preoperative variceal banding (Hahn et al. 2013)
3. Perform TEE after correcting coagulopathies (Hilberath et al. 2010)
4. Use rigid laryngoscope-assisted insertion of the probe to reduce the incidence of oropharyngeal mucosal injury and the number of insertion attempts (Na et al. 2009)
5. Abandon TEE examination if probe insertion or advancement is difficult (Kallmeyer et al. 2001)
6. Limit insertion of the probe to a midesophageal level (Aniskevich et al. 2010)
7. Avoid a wide range of probe tip flexion and unnecessary probe manipulation (Augoustides et al. 2006)
8. Avoid manipulation in a fixed flexion position (Augoustides et al. 2006)
9. Use a TEE probe with a temperature-control mechanism (Kharasch and Sivarajan 1996)
10. Place the TEE in “freeze” mode when not obtaining images (Kharasch and Sivarajan 1996)
11. Remove the TEE probe as soon as possible to limit the thermal and mechanical effects (Kharasch and Sivarajan 1996)

*TEE* transesophageal echocardiography

We acknowledge limitations of the study. With our observation limited to the perioperative period, late-presenting complications may contribute to a higher incidence of complications than what was reported here. Because of the retrospective study design, it was likely that only major complications such as esophageal perforation and massive hemorrhagic variceal rupture were documented in the charts. Minor hemorrhage, e.g., presence of blood-tinted secretions in the oropharynx, in the orogastric tube, or on the TEE probe upon removal likely were not documented. The study has a major risk of underreporting bias. Most patients with high-grade varices underwent preoperative banding, which may have lessened the risk of rupture. However, our review was underpowered to show the protective effect of variceal banding in preventing TEE-related variceal hemorrhage. Furthermore, TEE was not routinely used in all OLT cases. In patients without preoperative banding or with varices of unknown grade, TEE often was limited to midesophageal windows or was completely avoided. The decision to perform TEE was made by anesthesiologists, and potential bias in patient selection could not be eliminated. Future studies of prospective routine intraoperative TEE use are required to overcome these limitations.

Intraoperative TEE during OLT can provide valuable real-time data that may not be obtainable by other means during periods of potentially catastrophic hemodynamic instability, facilitating rapid reaction for proper intraoperative patient management. Our study suggests that intraoperative TEE was relatively safe to use, despite the risk of variceal hemorrhage. On the basis of our data and experience, we suggest intraoperative TEE for all OLT patients with a cardiac history and perioperative hemodynamic instability refractive to treatment. Critical information provided by TEE may facilitate more accurate fluid and hemodynamic management through direct observation of ventricular size, valve function, myocardial performance, and acute thrombotic events (Burtenshaw and Isaac 2006; Hofer et al. 2004). Performing TEE examinations in all OLT surgeries may be a challenge due to the availability of the TEE equipment and TEE trained anesthesiologists in some institutions. TEE operators still face the same potential complications of esophageal perforation, esophageal tear, hematoma, laryngeal palsy, dysphagia, dental injury, and death(Practice guidelines for perioperative transesophageal echocardiography. An updated report by the American Society of Anesthesiologists and the Society of Cardiovascular Anesthesiologists Task Force on Transesophageal Echocardiography 2010; Hilberath et al. 2010; Piercy et al. 2009). The risks and benefits should be carefully considered. The anesthesia and surgical teams should be ready to treat possible complications associated with TEE insertion.

## Conclusions

Concerns about esophageal variceal rupture in patients with coagulopathy may deter anesthesiologists from using intraoperative transesophageal echocardiography (TEE) during orthotopic liver transplantation. A retrospective review of 232 patients with documented esophagogastric varices and coagulopathy showed TEE was relatively safe, with a low incidence of major hemorrhagic complications.

## Methods

After obtaining approval from the Mayo Clinic Institutional Review Board in November 2011, we conducted a retrospective chart and TEE video review of all OLT cases at Mayo Clinic (Jacksonville, Florida, USA) from January 2003 through December 2013. Patients were excluded if they did not undergo intraoperative TEE placement or if they had incomplete medical records regarding use of the TEE probe.

Pre-surgical esophagogastroduodenoscopy (EGD) was performed to determine the severity of the esophageal varices and the history of endoscopic variceal band ligation. In patients with multiple EGD examinations, the most recent report before the OLT was analyzed. Esophageal varices were graded by gastroenterologists as absent, grade I (small, straight varices <5 mm), grade II (enlarged, tortuous varices <5 mm, occupying less than one-third of the lumen), and grade III (large, coil-shaped varices >5 mm occupying more than one-third of the lumen); grading was determined with the esophagus insufflated (Peck-Radosavljevic et al. 2005; Garcia-Tsao et al. 2007). The history of prior upper gastrointestinal bleeding in relation to intraoperative variceal bleed also was evaluated.

Omniplane TEE probes (Philips Model 21378A and 21369A) were used. The attending anesthesiologists performed probe placements and examinations during OLT. An orogastric tube was inserted to empty the stomach and then removed before insertion of the TEE probe. If blind insertion of the TEE probe was not successful, the general approach was to insert the probe using direct or video laryngoscopy. TEE probe tip position was limited to midesophageal views (for monitoring volume status, ventricular contractility, and valvular function) or were advanced to a transgastric view as part of a full evaluation. The transgastric view was omitted in patients with a history of varices because the distal 2–5 cm of the esophagus is the most common site of varices (Sharara and Rockey 2001). It was also a clinical decision, made by the anesthesiologists, to not advance the TEE probe in patients without a history of varices. The number of TEE procedures with limited midesophageal views vs a full examination with transgastric views were recorded. The TEE probe was put in “freeze” mode when not obtaining images and was removed at the end of surgery, before leaving the operating room.

Electronic record laboratory data for prothrombin time (PT), partial thromboplastin time (PTT), international normalized ratio (INR), platelet counts, and fibrinogen counts immediately before TEE probe insertion were reviewed. Data are presented as mean (SD), median, and interquartile range. We evaluated any major perioperative complications to determine whether TEE usage was associated with the severity of esophageal varices and coagulation status. Documentations by physicians and nursing staffs from the time in the operating room to 24 h postoperatively were reviewed for evidence of TEE-related complications. Complications were defined as dental injury (chipping or loss of teeth), oropharyngeal trauma (mucosal tear in the oropharyngeal area), esophageal or gastric mucosa injury (tissue tear confirmed by a gastroenterologist via upper endoscopy), or variceal hemorrhage (variceal rupture confirmed by a gastroenterologist via upper endoscopy).

## Abbreviations

EGD: esophagogastroduodenoscopy
INR: international normalized ratio
LVOTO: left ventricular outflow tract obstruction
OLT: orthotopic liver transplantation
PT: prothrombin time
PTT: partial thromboplastin time
TEE: transesophageal echocardiography
